# Serological and Molecular Survey on Domestic Dog Hepadnavirus in Household Dogs, Italy

**DOI:** 10.3390/ani13040729

**Published:** 2023-02-17

**Authors:** Paola Fruci, Andrea Palombieri, Vittorio Sarchese, Giovanni Aste, Klaus G. Friedrich, Vito Martella, Barbara Di Martino, Federica Di Profio

**Affiliations:** 1 University Veterinary Teaching Hospital, Department of Veterinary Medicine, University of Teramo, 64100 Teramo, Italy; 2 Fondazione Bioparco, Viale del Giardino Zoologico 20, 00197 Rome, Italy; 3 Laboratory of Infectious Diseases, Department of Veterinary Medicine, University of Bari Aldo Moro, 70010 Bari, Italy

**Keywords:** hepadnavirus, dogs, DCHCAbs, DCHSAbs, viral DNA

## Abstract

**Simple Summary:**

Hepadnaviruses, similar to human hepatitis B virus (HBV), have recently been discovered in several mammalian species, including domestic carnivores. Circulation of hepadnaviruses in cats (domestic cat hepadnavirus, DCH) has been documented in a number of molecular surveys, demonstrating a worldwide distribution. In contrast, studies on hepadnavirus epidemiology in dogs are still limited. In this study, we screened an age-stratified collection of 600 canine sera for the presence of antibodies against the recombinant DCH core antigen (DCHCAg) and DCH surface antigen (DCHSAg). DCHC antibodies were found with an overall prevalence of 10.0% (60/600). Out of 60 positive serum samples, 30.0% (18/60) also possessed IgG anti-DCHSAg. All samples were also assessed molecularly using a pan-hepadnavirus nested-PCR and a DCH-specific quantitative PCR to investigate the presence of viral DNA, revealing a prevalence rate of 0.7% (4/600). Our results demonstrated unequivocally that hepadnaviruses genetically and antigenically related to DCH circulate actively in the canine population, albeit with a lower prevalence than in cats.

**Abstract:**

The discovery of hepadnaviruses in cats (domestic cat hepadnavirus, DCH) and of a DCH-like virus in dogs has raised several questions regarding the role of these viruses in pets, with particular emphasis on their potential impact on animal health and epidemiology, as well as possible zoonotic implications. In this study, by screening an age-stratified collection of 600 canine serum samples for DCH with an ELISA assay based on the recombinant core antigen (DCHCAg), specific antibodies were found with an overall prevalence of 10.0% (60/600), with a higher prevalence in younger and older dogs. By retesting the canine DCHCAbs-positive sera with an ELISA test based on the recombinant surface protein of DCH (DCHSAg), a total of 18 sera (30%, 18/60) also contained IgG anti-DCHSAg. All the sera were also assessed molecularly using either a consensus hepadnavirus PCR or a specific real-time PCR for DCH. Hepadnavirus DNA was detected in four seronegative dogs, with a prevalence rate of 0.7% (4/600). On sequence analysis of the polymerase region amplified with pan-hepadnavirus primers, the amplicons displayed the highest nucleotide identity (97.3–99.6%) to DCH sequences detected in cats and to the domestic dog hepadnavirus recently identified in a canine serum sample from Italy.

## 1. Introduction

The *Hepadnaviridae* family comprises small, enveloped viruses with a partially double-stranded circular DNA genome of 3.0–3.4  kb in size, organized in four overlapping open reading frames including surface (S), core (C), polymerase (P), and X genes [1]. According to the classification criteria of the International Committee on Taxonomy of Viruses (https://www.ictv.global/report/hepadnaviridae, accessed on 15 December 2022), the family is currently divided in five different genera: *Parahepadnavirus*, *Metahepadnavirus*, *Herpetohepadnavirus*, *Avihepadnavirus*, and *Orthohepadnavirus* [2]. Only members of the genus *Orthohepadnavirus* infect mammals, and the prototype species, hepatitis B virus (HBV), is an important and globally distributed viral pathogen causing around 820,000 deaths annually, mostly as a consequence of cirrhosis and hepatocellular carcinoma (HCC) [3].

In recent years, taking advantage of massive sequencing technologies, close relatives of HBV have been detected in a variety of mammalian species [4,5,6,7,8,9], including domestic cats and dogs [10,11,12]. First evidence on the susceptibility of a domestic carnivore to hepadnavirus (domestic cat hepadnavirus, DCH) infection was documented in Australia in 2018 [10] during a transcriptomic study aimed at analyzing alterations in gene expression in a 7 year old domestic shorthair cat with multicentric large B-cell lymphoma and feline immunodeficiency virus (FIV) infection [10]. Subsequent molecular and serological investigations performed in Australia [10], Italy [13,14], Thailand [15], Malaysia [16], Japan [17], and the United States [18] have revealed that DCH is broadly circulating in domestic cat populations worldwide. Although its pathogenic potential is still uncertain, a positive correlation between an increased level of markers indicative of structural or functional liver damage and high serum viral loads (>10^4^ genome copies per mL) was observed [13,16]. Furthermore, histologic features of inflammation and neoplasia similar to those caused by HBV have been found in the liver of cats with DCH-associated chronic hepatitis or HCC [19].

In recent years, using a real-time PCR (qPCR) based on specific primers for DCH [13] hepadnavirus DNA was detected in 6.3% (40/635) of canine sera in a molecular investigation conducted in Italy [12]. On complete genome sequencing of one strain (570/ITA, GenBank accession no. MZ201309), the virus displayed 98.0% nucleotide (nt) identity to the Italian DCH strain ITA/2018/165-83 (MK117078) [13] and 96.9% nt identity to the Australian DCH reference strain Sydney2016 (MH307930) [10], respectively. Further evidence on the possible susceptibility of dogs to DCH has also been obtained in a molecular investigation conducted in Hong Kong [20], in which viral DNA was detected in 0.4% (2/501) of canine blood samples tested. Although the virus seems more common in dogs with altered hepatic markers [12], no evidence supporting its involvement in canine liver diseases was found when assessing 101 biopsies from dogs with idiopathic chronic hepatitis and HCC in Hong Kong or the USA [20].

Before the identification of a DCH-like virus in dogs [12,20], direct virological demonstration on the circulation of hepadnaviruses in the canine population was reported in a study performed in Brazil in 2019 [11], in which 5.8% (11/189) of sera collected from stray dogs were found to be positive for the presence of the HBV surface antigen (HBSAg). Furthermore, using two set of primers targeting the preS/S1 genomic region and the core gene of HBV, viral DNA was detected in 10.0% of dog sera tested. In the partial S gene (about 1 kb) of one such strain (GenBank accession no. MF991935), the newly identified canine hepadnavirus shared 84.8–98.4% nt identity to sequences detected in bile samples of domestic pigs and in sera from HBV patients and nonhuman primates, while the identity to the Australian DCH reference strain Sydney2016 was 64.4%.

Although epidemiological studies are still limited, preliminary data seem to indicate that dogs can host genetically diverse hepadnaviruses. However, whether these viruses are common in domestic dogs or they were detected serendipitously is still unclear. Herein, to draw a more complete picture of hepadnavirus epidemiology in dogs, we report the results of a serological screening performed on a collection of 600 household canine sera, based on the use of a DCH recombinant core antigen (DCHCAg) and surface antigen (DCHSAg). All samples were also assessed molecularly using a pan-hepadnavirus nested-PCR [6] and a DCH-specific qPCR [13] to investigate the presence of viral DNA.

## 2. Materials and Methods

### 2.1. Sampling

Serological and molecular screenings for hepadnaviruses were performed on 600 serum samples collected between January 2018 and July 2022 from overtly healthy household dogs admitted to the laboratory of the Veterinary Teaching Hospital at the Department of Veterinary Medicine (University of Teramo, Italy) for pre-surgical evaluation. The samples were divided into six age groups: <1 year (*n* = 106), 1–3 years (*n* = 64), 4–8 years (*n* = 158), 9–11 years (*n* = 135), 12–14 (*n* = 99), and >15 years of age (*n* = 38). Informed consent was obtained from all dog owners.

### 2.2. Expression of DCHSAg in the Baculovirus System and Development of Antibody Detection Enzyme-Linked Immunosorbent Assay (ELISA)

**Figure 1:**
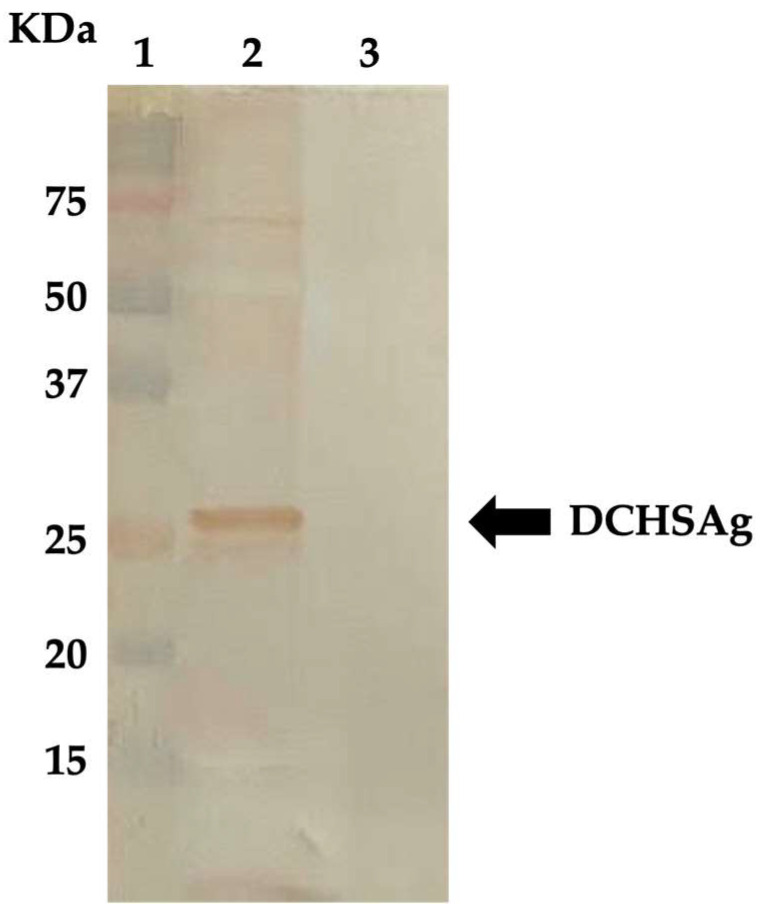
WB analysis of the DCHS protein using a feline serum that previously resulted positive for IgG anti-DCHC. Line 1: Precision Plus protein standards; line 2: recombinant DCHS protein; line 3: mock-infected *Sf9* insect cells. The arrow indicates the band of positivity.

The recombinant baculovirus vector carrying the surface (S) protein-encoding gene (681 nt and 226 aa in length) of the DCH strain ITA/2018/165-83 (GenBank accession no. MK117078) [13] was synthesized as previously described [14]. For large-scale production of the S protein, 100 mL of *Spodoptera frugiperda* (*Sf9*) cells (1 × 10^6^ cell/mL) suspension culture were infected with the recombinant baculovirus at a multiplicity of infection of three plaque-forming units/cell. The S protein was isolated from the culture medium of infected cells by centrifugation at 4000 rpm for 20 min. After purification by rate-zonal centrifugation on a discontinuous 20–60% (wt/vol) sucrose gradient, the collected fractions were dialyzed against PBS, and the protein concentration was determined by measuring the optical density at 280 nm (OD_280_). A band with a molecular weight of ~25.4 kDa corresponding in size to the DCH S protein was observed in the supernatant of *Sf9* insect cells from 5 days post infection, using SDS-12% polyacrylamide gel electrophoresis (PAGE) and Coomassie brilliant blue staining. The immunogenicity of the DCH S protein was evaluated by Western blotting (WB) (Figure 1) using a selection of cat sera previously found positive for IgG antibodies against the DCHCAg [14], but negative for the presence of hepadnavirus DNA. The WB was performed following the same procedure previously described [14]. Briefly, the purified surface protein was dissolved in sample buffer (375 mM Tris–HCl, 60% glycerol, 12% SDS, 0.6 M DTT, 0.06% bromophenol blue) and denatured for 5 min at 95 °C prior to electrophoresis. The protein, after characterization by a sodium dodecyl sulfate polyacrylamide gel electrophoresis (SDS-PAGE) using 12% gels run in a Mini-Protean Tetra Vertical Electrophoresis Cell (Bio-Rad Laboratories, Milan, Italy), was transferred to Immobilon^®^-P membrane (Millipore, Milan, Italy) via semi-dry transfer for 1 h at 100 mA. After electroblotting, the membrane was blocked with SuperBlock™ Blocking Buffer (Invitrogen, Ltd., Paisley, UK) and incubated for 1 h with gentle agitation. The blot was placed in a diluted feline serum (final dilution of 1:100) and then incubated for 1 h at room temperature. After washing five times with 0.1% Tween/phosphate-buffered saline (PBS-T), the blot was incubated with horseradish peroxidase-conjugated goat anti-cat IgG (Sigma-Aldrich, Milan, Italy) at dilution of 1:3000 for 1 h. The reaction was developed by adding DAB substrate solution (3,3′-diaminodbenzidine) (Sigma-Aldrich, Milan, Italy) enriched with 12.5 µL of hydrogen peroxide.

For the development of the enzyme-linked immunosorbent assay (ELISA), mock infected *Sf9* insect cell lysate and the recombinant DCHSAg, at the final concentrations of 1 µg/mL, were coated onto 96-well EIA plates (Costar, Milan, Italy) at 100 µL per well in carbonate–bicarbonate buffer (0.05 M, pH 9.6). The wells were washed five times with 0.1% Tween/phosphate-buffered saline (PBS-T), and then 200 µL of SuperBlock™ Blocking Buffer (Invitrogen, Ltd., Paisley, UK) was added and incubated at room temperature for 2 h. To determine the optimal working serum dilutions, twofold dilutions of dog sera positive for IgG anti-DCHSAg in WB were prepared starting with a dilution of 1:25 until 1:400. Each serum dilution was added to wells coated with either DCHSAg or mock infected *Sf9* cells. Plates were washed five times with 0.1% PBS-T and then incubated with horseradish peroxidase-conjugated goat anti-dog IgG (Sigma-Aldrich, Milan, Italy) at a dilution of 1:5000 for 30 min at 37 °C. The reaction was developed by adding 100 µL/well of 2,2′-azino-di-(3-ethylbenzthiazoline-6-sulfonate) (ABTS) (Invitrogen, Ltd., Milan, Italy) substrate incubated at room temperature for 15 min. The serum dilution, established as 1:100, was considered optimal when, for each serum tested, a positive/negative ratio (OD_405_ of DCH antigen/OD_405_ of mock infected cells) ≥2.0 was obtained. The cutoff point of the ELISA (OD_405_ ≥ 0.87 for IgG) was determined as the mean of the OD_405_ readings (ranging from 0.38 to 0.86) of 15 dog serum samples negative in WB for the presence of IgG anti-DCHSAg plus two standard deviations.

### 2.3. Serological Screening for Anti-DCHCAg and Anti-DCHSAg

An in-house ELISA assay, based on the recombinant C protein of the DCH strain ITA/2018/165-83 [13] previously expressed in the baculovirus system [14], was used with some modifications to evaluate, in the 600 dog sera, the presence of IgM or IgG anti-DCHCAg. Briefly, after coating of DCHCAg onto a 96-well EIA plates (Costar, Italy) and subsequent blocking, each dog serum at dilution of 1:100 was added to wells coated with either DCHCAg or mock infected *Sf9* cells. After five washes with 0.1% PBS-T, plates were incubated with horseradish peroxidase-conjugated goat anti-dog IgM (Bio-Rad, Italy) or goat anti-dog IgG (Sigma-Aldrich, Milan, Italy) at the dilution of 1:10,000 and 1:5000, respectively, for 30 min at 37 °C. The cutoff points of the ELISA were established as OD_405_ ≥ 0.7 for IgM (mean OD_405_ readings of 25 dog sera negative in WB ranging from 0.2 to 0.69, plus two standard deviations) and OD_405_ ≥ 0.8 for IgG (mean OD_405_ readings of 25 dog sera negative in WB ranging from 0.3 to 0.79, plus two standard deviations). All seropositive specimens were further tested to evaluate seroconversion against DCHSAg. Serological screening for the presence of anti-DCHSAg was also performed on a subset of canine serum samples negative for antibodies anti-DCHCAg. For each sera sample, ELISA assays were repeated twice, confirming all positive results in WB. Differences in prevalence among the age groups and between serologic and molecular results were analyzed using Fisher’s exact test. A significance level of 5% was assumed for the test (*p*-value < 0.05). Statistical analysis was conducted in GraphPad Prism Software v. 9.5.1 (https://www.graphpad.com/scientific-software/prism/, accessed on 5 November 2022).

### 2.4. Molecular Screening for Hepadnavirus

Total DNA was extracted from 200 μL of each serum using the ExgeneTM Viral DNA/RNA mini (TEMA Ricerca, Bologna, Italy), following the manufacturer’s instructions. The presence of viral DNA was evaluated using a pan-hepadnavirus nested PCR able to amplify a 258 nt fragment of the polymerase ORF [6]. Amplicons of expected size were purified using the QIAquick gel extraction kit (Qiagen GmbH, Hilden, Germany). Each fragment was directly sequenced using a BigDye Terminator Cycle chemistry and 3730 DNA Analyzer (Applied Biosystems, Foster, CA, USA). The Basic Local Alignment Search Tool (BLAST; http://www.ncbi.nlm.nih.gov, accessed on 1 December 2022) and FASTA (http://www.ebi.ac.uk/fasta33, accessed on 1 December 2022) with default values were used to find homologous hits. For quantification of the viral loads, all specimens were also assessed by specific qPCR for DCH, targeting a 105 nt fragment of the polymerase region, following the procedure previously described in [13,14].

## 3. Results

### 3.1. Detection of Antibodies Anti-DCHCAg (DCHCAbs)

Out of the 600 dog samples tested at the dilution of 1:100, a total of 60 (10.0%) were found positive for the presence of DCHCAbs (Table 1). Of these, 45 (7.5%) reacted only for IgG, with OD_405_ values ranging from 0.8 to 1.9 (mean OD_405_ of 1.15); 13 sera (2.2%) were positive only for IgM (mean OD_405_ of 0.93), whilst two (0.3%, 2/600) possessed both IgM and IgG (mean OD_405_ of 1.1 and 1.2, respectively).

**Figure 2:**
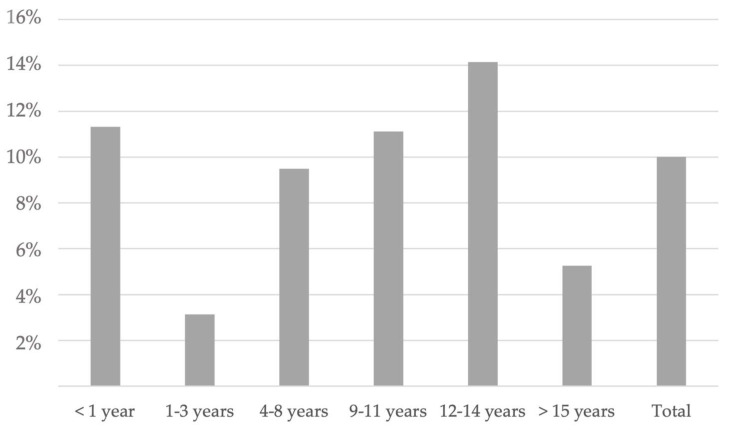
Distribution of total DCHCAbs among different age groups.

We further analyzed the seroprevalence age-related pattern combining IgM and IgG positivities (Figure 2).

The seropositive rate of total DCHCAbs was 11.3% (12/106) in puppies <1 year of age and 3.1% (2/64) in dogs of 1–3 years of age, followed by a gradual increase in the positive rates from 9.5% (15/158) in the 4–8 year age group to 11.1% (15/135) and to 14.1% (14/99) in the 9–11 and 12–14 year age groups, respectively. DCHCAbs were detected in dogs older than 15 years with a rate of 5.3% (2/38). A significant difference in DCHCAbs distribution by age classes was observed only when comparing the age groups of 1–3 years and 12–14 years (*p* = 0.028), whereas there were no statistically significant differences comparing the seroprevalences among the other age groups (*p* > 0.05).

### 3.2. Molecular Detection and Sequence Analysis

On molecular screening using pan-hepadnavirus primers, viral DNA was detected in four serum samples, with an overall prevalence of 0.7% (4/600). None of the four viremic dogs possessed DCHCAbs. The same sera resulted positive when retested by specific qPCR for DCH [13], with viral loads ranging from 1.7 × 10^1^ to 1.5 × 10^2^ DNA copies/5 μL of template (mean 3.4 × 10^1^ DNA copies). On sequence analysis of the 258 nt amplicons obtained with pan-hepadnaviridae primers [6], the four strains displayed nt identities of 97.7–100% to each other, with the highest (97.3–99.6% nt identity) to DCH sequences currently available on GenBank database. Identity to the domestic dog hepadnavirus (GenBank accession no. MZ201309) recently detected in a canine serum sample from Italy [12] was 97.5–98.9%.

### 3.3. Detection of Antibodies Anti-DCHSAg

All the sera possessing either IgM or IgG for DCHCAg were assessed for the presence of IgG anti-DCHSAg, revealing an overall prevalence of 30.0% (18/60). In detail, IgG anti-DCHSAg were detected in 17 (37.7%) of the 45 sera with IgG anti-DCHCAg, at a dilution of 1:100, with OD_405_ values ranging from 0.87 to 1.6 (mean OD_405_ of 1.1) but in none of 13 sera with IgM anti-DCHCAg. Seroconversion against the DCHSAg was also observed in one of the two sera reacting with both IgG and IgM anti-DCHCAg. When retesting a subset of 159 seronegative specimens for DCHCAbs, none of the tested sera showed seroconversion against DCHSAg.

## 4. Discussion

Investigating the patterns and phases of HBV infection is challenging and requires analysis of multiples targets (antibodies, antigens, and DNA) [21,22]. Furthermore, investigating the patterns of hepadnavirus infection in cats and dogs cannot rely solely on molecular diagnostics. A recombinant DCHCAg produced in a baculovirus expression system was used to develop an antibody detection ELISA to study DCH seroprevalence in a population of household cats. Of 256 cats tested, 64 (25%) were positive for anti-DCHC whist only 25 cats (9.8%) tested positive for DCH DNA, demonstrating that exposure of cats to DCH is 2.5 times higher when assessed with indirect diagnostics. DCHCAbs in feline sera may be considered a serologic marker indicating past exposure or current DCH infection [14].

In the present study, an in-house DCHCAbs-ELISA was employed to screen 600 sera collected from a population of household dogs randomly selected among animals hospitalized for surgery, revealing an overall seroprevalence of 10.0% (60/600). The same prevalence rate was reported in a study in Brazil [11], in which total HBCAbs were detected in 19 (10.0%, 19/189) Brazilian free-roaming dogs.

By analyzing the seropositivity age-related pattern in our sera, a prevalence rate of 11.3% (12/106) was observed in puppies (<1 year). This rate decreased to 3.1% (2/64) in dogs of 1–3 years and steadily increased with age reaching 14.1% (14/99) in animals of 12–14 years of age. This U-shaped curve could indicate higher susceptibility or risk of exposure for younger and older animals. In our study, a substantial percentage of seropositive dogs contained only IgG anti-DCHCAg (75.0%, 45/60), whilst IgM were found alone or in conjunction with IgG, respectively, in 21.6% (13/60) and 3.3% (2/60) of the positive sera. In HBV infection, IgM anti-HBCAg is often considered to be a precise marker for the differential diagnosis between acute (persisting for up to 6 months) and chronic infection [23]. Making parallelism with anti-HBC antibodies dynamic, an acute phase of hepadnavirus infection may be hypothesized for the cohort of IgM-positive dogs. We also assessed the circulation of hepadnaviruses in dogs molecularly, using a nested PCR approach based on primers targeting highly conserved motifs of the polymerase gene in the family *Hepadnaviridae* [6] and a qPCR specific for DCH [13]. Viral DNA was identified in four serum samples with an overall prevalence of 0.7% (4/600), which is slightly higher than the rate (0.4%, 2/501) recently reported in Hong Kong [20], but lower than the prevalence rates reported in surveys in Brazil (10.0%, 19/189) and Italy (6.3%, 40/635) [11,12]. These variations rates may reflect geographical patterns or the study design, i.e., the target canine populations [12]. For instance, the dogs assessed in this study were all apparently healthy, whilst, in the study of Diakoudi et al. [12], sampling comprised different subsets of dogs, including animals with altered hepatic markers or immunosuppressive infections.

In spite of several attempts, we were not able to reconstruct the complete genome of the viruses identified in dogs in this study, due to the low viral load. However, on sequence analysis of the 258 nt fragment generated with the nested pan-hepadnavirus protocol, all the four amplicons displayed the highest identity (97.3–99.6% nt) to DCHS currently available on GenBank database, confirming previous findings that dogs and cats may share similar hepadnaviruses [12].

Overall, serological and molecular rates detected in dogs in our analysis were lower than those previously reported in cats (25.0% (64/256) and 9.8% (25/256), respectively) in a similar investigation performed in Italy [14]. A marked difference was also observed in terms of detectable viral load in the four viremic dogs, which was on average of 3.4 × 10^1^ DNA copies, whilst that in the feline sera resulted as high as 1.1 × 10^3^. A significant correlation between the presence of viral DNA and DCHCAbs was found in cats, with the majority of viremic animals (92.0%, 23/25) resulting also seropositive [14]. Herein, none of the viremic dogs possessed antibodies against DCHCAg; in turn, all seropositive animals, including those reacting with IgM, resulted negative when assessed with consensus or specific PCRs. Taken together, these findings may reflect a low susceptibility of dogs to this infection, with viral shedding at lower titers and for a shorter period than in cats. In human hepatitis B, the presence of HBCAbs as the only positive serum marker is detectable in approximately 40% of occult HBV infections [24], while concomitant seroconversion to anti-HBSAg indicates the recovery from infection, conferring a long-term protective immunity. In addition, HBSAb is the only marker in persons who successfully respond to hepatitis B vaccination. In this survey, by retesting all the canine DCHCAbs-positive sera with the ELISA based on the recombinant S antigen of DCH, we found that a total of 18 samples (30%, 18/60) also possessed IgG anti-DCHSAg, a condition that may be compatible with possible recovery from infection. In contrast, when we rescreened a subset of 159 dog DCHCAbs-negative sera, DCHSAbs were not found, suggesting that these animals likely had never been exposed to hepadnavirus infections. Interestingly, positivities in dogs for HBSAbs have already been documented in three studies performed in USA [25], Taiwan [26], and Iraq [27], revealing prevalence rates of 48.0%, 59.1%, and 9.0%, respectively. The significance of this seropositivity, together with that found for HBCAg [11], remains still unclear. Some authors [11,25,27] have hypothesized that dogs could be exposed to HBV from the environment contaminated by infected humans. Otherwise, the presence of anti-HBV antibodies in canine sera could be the result of serological cross-reactivities between HBV and other antigenically related hepadnaviruses. Cross-antigenic reactivity has recently been found for DCHCAg, which was identified in feline tissues by immunostaining [15] using a polyclonal serum specific for HBV core protein. These findings, together with the direct identification of DCH-like viruses in dogs [12,20] and data collected in the present survey, make feasible the hypothesis that the HBV-specific antibodies previously detected in dogs [11,25,27] could be due to exposure to DCH-like viruses infecting domestic carnivores rather than exposure to HBV.

## 5. Conclusions

The results of this study demonstrated unequivocally that hepadnaviruses genetically and antigenically related to DCH circulate actively in the canine population, albeit with a lower prevalence than in cats. Whilst there is some evidence for an etiopathogenetic role of DCH in the development of feline liver disease [13,15,16,19], the clinical impact of DCH in dogs, if any, is still unclear and warrants further studies in canine populations. Coupling direct and indirect investigations is necessary to provide depth and dimensions to the study of DCH in carnivores.

## Figures and Tables

**Table 1:** Seroprevalence of DCHCAbs (IgM and IgG) in dog serum specimens.

DCHCAbs	Positive	Negative
**IgM anti-DCHCAg**	13 (2.2%)	587 (97.8%)
**IgG anti-DCHCAg**	45 (7.5%)	555 (92.5%)
**IgM and IgG anti-DCHCAg**	2 (0.3%)	598 (99.7%)
**Total**	60 (10.0%)	540 (90.0%)

## Data Availability

The data presented in this study are available in the manuscript.
